# P2X_7_ signaling promotes microsphere embolism-triggered microglia activation by maintaining elevation of Fas ligand

**DOI:** 10.1186/1742-2094-9-172

**Published:** 2012-07-12

**Authors:** Ying-mei Lu, Rong-rong Tao, Ji-yun Huang, Li-tao Li, Mei-hua Liao, Xiao-ming Li, Kohji Fukunaga, Ze-Hui Hong, Feng Han

**Affiliations:** 1 Institute of Pharmacology, Toxicology and Biochemical Pharmaceutics, Zhejiang University, 866 Yu-Hang-Tang Road, Hangzhou, 310058, China; 2 Department of Neurobiology, Key Laboratory of Medical Neurobiology of Ministry of Health of China, Zhejiang University School of Medicine, Hangzhou, China; 3 Department of Pharmacology, Graduate School of Pharmaceutical Sciences, Tohoku University, Sendai, Japan; 4 The Key Laboratory of Developmental Genes and Human Disease, Institute of Life Science, Southeast University, Nanjing, China

**Keywords:** Microsphere embolism, Microglia, P2X_7_ receptor, Fas ligand, Neuronal cell death

## Abstract

**Background:**

The cerebral microvascular occlusion elicits microvascular injury which mimics the different degrees of stroke severity observed in patients, but the mechanisms underlying these embolic injuries are far from understood. The Fas ligand (FasL)-Fas system has been implicated in a number of pathogenic states. Here, we examined the contribution of microglia-derived FasL to brain inflammatory injury, with a focus on the potential to suppress the FasL increase by inhibition of the P2X_7_-FasL signaling with pharmacological or genetic approaches during ischemia.

**Methods:**

The cerebral microvascular occlusion was induced by microsphere injection in experimental animals. Morphological changes in microglial cells were studied immunohistochemically. The biochemical analyses were used to examine the intracellular changes of P2X_7_/FasL signaling. The BV-2 cells and primary microglia from mice genetically deficient in P2X_7_ were used to further establish a linkage between microglia activation and FasL overproduction.

**Results:**

The FasL expression was continuously elevated and was spatiotemporally related to microglia activation following microsphere embolism. Notably, P2X_7_ expression concomitantly increased in microglia and presented a distribution pattern that was similar to that of FasL in ED1-positive cells at pathological process of microsphere embolism. Interestingly, FasL generation in cultured microglia cells subjected to oxygen-glucose deprivation-treated neuron-conditioned medium was prevented by the silencing of P2X_7_. Furthermore, FasL induced the migration of BV-2 microglia, whereas the neutralization of FasL with a blocking antibody was highly effective in inhibiting ischemia-induced microglial mobility. Similar results were observed in primary microglia from wild-type mice or mice genetically deficient in P2X_7_. Finally, the degrees of FasL overproduction and neuronal death were consistently reduced in P2X_7_^−/−^ mice compared with wild-type littermates following microsphere embolism insult.

**Conclusion:**

FasL functions as a key component of an immunoreactive response loop by recruiting microglia to the lesion sites through a P2X_7_-dependent mechanism. The specific modulation of P2X_7_/FasL signaling and aberrant microglial activation could provide therapeutic benefits in acute and subacute phase of cerebral microembolic injury.

## Introduction

Epidemiological and clinical evidence has indicated that cerebral microvascular occlusion is a common phenomenon throughout life, which might require better understanding as a mechanism of ischemic insult and subsequent neuronal damage [1-3]. Unlike the lethal larger artery occlusion, microsphere embolism elicits microvascular injury which mimics the different degrees of stroke severity observed in patients [2,4-6], but the mechanisms underlying these embolic injuries are far from understood. Cerebral ischemia is followed by destruction of microvasculature and subsequent inflammatory events during acute and subacute stages of ischemia [3,7,8]. It is noteworthy that there is a growing body of evidence that uncontrolled inflammatory response in the brain can contribute to exacerbate the neuronal loss in cerebral ischemia [3,7,8]. The spatiotemporal changes in microglial dynamics are dramatically affected by capillary blood flow around the microglial somata in intact ischemic mouse neocortex [9]. However, no link between brain inflammatory response and cerebral microvascular occlusion has been studied thus far.

The Fas ligand (FasL)-Fas system may play a role in both physiological and pathological situations in the brain [10,11], whether and how FasL-Fas system influences the pathogenesis of microembolic injuries remains unknown. In pathological conditions, dysregulated FasL-Fas system leads to activation of Fas-associated death domain (FADD), caspase 8, and subsequently neuronal death [12,13]. By contrast, depending on the cell type and its state, FasL-Fas system has also been shown to be instructive signal for ongoing and injury-induced brain regeneration [10]. Despite the autocrine action of FasL that has been shown in vascular smooth muscle cells [14], epithelial cells [15], lymphocytes [16], and intestinal cells [17], little is known about its effects on microglia or its possible role in brain microembolic injury. Due to the dual and insufficient knowledge about FasL-Fas system in the brain, discrete targets amenable to ischemic processes need to be identified and functionally validated.

Microglial cell activation is a rapidly occurring cellular response to cerebral ischemia, which is a strictly controlled event [18-22]. Activated microglia may exert a cytotoxic effector function by stimulating morphological changes and the production of a wide array of inflammatory cytokines, which may cause bystander damage following ischemia [18,21]. The mechanisms by which microglia are activated during the acute and subacute phase of brain ischemia, or by which microglia continue to synthase or secrete FasL remain unknown. The exposure of microglia to H_2_O_2_ or paraquat rapidly triggers FasL mRNA transcription and protein expression and also activates NF-kappa B [23,24]. However, activated microglia under certain circumstances may act on different steps in the formation, maturation, and functional integration of the new neurons of both intact and pathological brain [25]. The opposing neurotoxic and neuroprotective properties of microglia during ischemic injury provide a rich and currently unexplored set of research problems.

The present study investigated the pathophysiological relevance of cerebral microembolic injury-induced microglial activation, FasL intracellular signaling, and the neuronal death cascade. We examined the influence of ischemic injury on microglia cells as well as the functional outcome of aberrant FasL signaling *in vitro* and *in vivo*. Herein, we report that the activation of P2X_7_ in microglia results in a gradual accumulation and release of FasL along with the subsequent activation of downstream cascades in ischemic context.

## Materials and methods

### Reagents

All chemicals were purchased from Sigma-Aldrich Chemical Co. (St Louis, MO, USA) unless otherwise noted.

### Mouse strains

Homozygous P2X_7_^−/−^ mice (B6.129P2-*P2rx7*^*tm1Gab*^/J, Jackson Laboratories, Bar Harbor, ME, USA), *gld/gld* mice (C3H/HeJ-Jms, FasL-deficient, SLC, Japan), and wild-type (WT) littermates of both backgrounds were used for the experiments. All of the animal procedures conformed to appropriate guidelines for the care and use of laboratory animals and were approved by the Committees for Animal Experiments of Zhejiang University in China and Tohoku University in Japan.

### Cerebral microemboli model

The microsphere embolism model was prepared as previously described [5]. Briefly, male Wistar rats weighing 220 g to 270 g were subjected to general anesthesia, after which 1,000 non-radioactive microspheres (48.4 ± 0.7 μm in diameter) were injected into the left carotid artery. The rats were decapitated 2, 6, 12, 24, 72, or 168 h after microsphere injection, and the ipsilateral cerebral hemisphere was dissected for further analysis. For P2X_7_^−/−^ mice, *gld/gld* mice, and their respective WT littermates, 5,000 non-radioactive microspheres (15 μm in diameter) were injected into the left carotid artery. Neurological scores were assessed 168 h after microsphere embolism as previously described [5].

### MicroPET scans

2-[18^F^-fluoro-2-deoxy-D-glucose (18^F^-FDG) injections and microPET scans were processed as previously reported [26]. The data of 18^F^-FDG microPET imaging were acquired from rats 168 h after microsphere embolism by using a microPET R4 scanner (Concorde Microsystems, Knoxville, TN, USA).

### Drug treatment

Drug administration began after a 1-day recovery period following microsphere embolism in rats. Minocycline hydrochloride (Sigma-Aldrich) (45 mg/kg, dissolved in distilled water) was intraperitoneally administered daily for 6 consecutive days. The ipsilateral hemispheres were then dissected for western blot analysis.

### Oxygen-glucose deprivation and neuron-conditioned medium

Cortical neurons were prepared from E18 C57BL/6 mouse embryo cortices as previously described [27]. The mice were decapitated to minimize pain and distress. Dissection and dissociation were carried out in 1× Hank’s balanced salt solution (HBSS) and 0.25% trypsin (Gibco) for 10 min before being terminated with 1× HBSS and 10 mg/mL trypsin inhibitor. Neurons were also dissociated by trituration in HBSS containing DNase I with a flame-narrowed pasteur pipette to fully dissociate the cells. Neurons were seeded at an approximate density of 2 × 10^7^ on 6-cm dishes (430166, Corning) and maintained in 5 mL of neurobasal medium containing B27 supplement (2%, Invitrogen), penicillin-streptomycin (100 U/mL penicillin, 100 μg/mL streptomycin, Gibco) and 0.25% GlutaMAXTM-1 (Gibco). Neurons were grown *in vitro* for 7 days.

To create an *in vitro* ischemia-like injury model, the primary cortical neurons were subjected to oxygen-glucose deprivation (OGD). The procedure for OGD challenge was performed as previously reported [6]. The medium was removed from the cultures, and the cells were rinsed twice with glucose-free HBSS prior to being subjected to OGD at defined time points. The OGD-treated cell-free conditioned medium (OCM) was collected to perform related analyses.

### BV-2 murine microglial cells culture and migration assay

BV-2 murine microglial cells, which exhibit similar morphological and functional properties comparable to primary microglial cells [28,29], were grown in Dulbecco’s modified Eagle’s medium supplemented with 10% fetal bovine serum at a density that did not exceed 5 × 10^5^ cells/mL. To mimic ischemia-like injury, the BV-2 immortalized mouse microglial cell line was subjected to OCM. Migration was assessed with a transwell migration chamber as described in a previous report [30]. Briefly, BV-2 murine microglial cells were placed in a transwell migration chamber and cultured in DMEM medium supplemented with 10% fetal bovine serum (FBS) at a density of 2 × 10^5^ cells/mL. The cells were allowed to migrate through a polycarbonate mesh (8.0 μm pore size, polycarbonate membrane, Corning Costar Corp., Cambridge, MA, USA) at 37°C. For the experiments that used neutralizing antibody, the cells were preincubated with anti-mouse FasL neutralizing antibody (2 μg/mL, 8 μg/mL, or 16 μg/mL; Santa Cruz Biotechnology Inc., Santa Cruz, CA, USA, Catalog no: sc-19988) or control preimmune serum for 30 min before ATP or recombinant FasL treatment. Recombinant FasL (100 ng/mL, Sigma-Aldrich, Catalog no: F0552) was placed in the lower chamber to induce migration. The chamber was kept in 5% CO_2_ at 37°C for 24 h. Microglia that migrated to the lower surface were fixed with 4% paraformaldehyde and stained with the blue-fluorescent DAPI. The rate of microglial migration was calculated by counting the cells of each well using a 10× bright-field objective. The number of cells in each well was normalized to the average number of cells in the control condition (100%). The experiments were repeated at least three times.

### Lentiviral infection

To construct the P2X_7_ short hairpin RNA (shRNA) expression plasmid, two complementary P2X_7_ DNA oligonucleotides (see below) were synthesized, annealed, and inserted into the pMagic 4.1 plasmid. Reduction of P2X_7_ expression was achieved through lentiviral infection of BV-2 microglia with the P2X_7_ cDNA coding sequence 5’-GCAAGTTGTCAAAGGCCAA-3’, and the resulting culture supernatant was used for the lentiviral infection. As a control, microglia were infected in a similar manner with the shRNA sequence 5’-TTCTCCGAACGTGTCACGT-3’. The cells were collected for further experiments at 72 h postinfection.

### RNA interference of FasL expression

FasL siRNAs (Santa Cruz Biotechnology Inc., Santa Cruz, CA, USA) were introduced into BV-2 murine microglial cells with transfection medium according to the manufacturer’s instructions. The control set of BV-2 cells was transfected with non-targeted siRNAs. The cells were collected for experiments 24 h after transfection. Knockdown was confirmed with western blotting using whole cell lysates.

### ELISAs for extracellular Fas ligand

BV-2 murine microglial cells were either infected with lentivirus directing the expression of non-targeting control or P2X_7_-specific shRNAs or transfected with siRNAs against FasL. Supernatants from the OGD-treated cortical neurons were applied to BV-2 cultures for the measurement of the FasL level. Target cells were then cultured with OCM for 6 h, and Fas ligand assays were performed with a mouse Fas ligand/TNFSF6 immunoassay kit (R&D Systems, Minneapolis, MN, USA) following the manufacturer’s protocol.

### Primary microglial cell culture and chemotaxis assays

Primary mixed glial cultures were prepared according to a previously described method with slight adjustments [31]. Briefly, mixed glial cultures were prepared from the cerebral cortices of 1-day-old mice using 0.25% trypsin, and the cells were cultured in minimal essential medium (MEM) containing 10% FBS(Gibco) at 37°C in a 5% CO_2_ humidified incubator. After 10 to 14 days, the cultures were shaken by hand for 15 min, the floating cells were collected and centrifuged for 5 min at 1000 × g, and microglial cells were seeded on glass-bottom dishes (Matsunami, Osaka, Japan) at a density of 2 × 10^4^ cells per dish.

For the chemotaxis assays, the cells were exposed to a chemoattractant gradient that was generated by slowly releasing ATP from a micropipette tip that was placed at the center of the imaging field. Cell migration toward the pipette tip was monitored over a 30-min period by phase-contrast time-lapse microscopy. Acquisition was performed using an Olympus microscope with a × 20 air objective (LCACH, 0.4 NA), and the images were processed and analyzed using Fluoview software (FV10-ASW 5.0, Japan) [32].

### Western blotting

Proteins were extracted, and the soluble cytosolic fraction was isolated according to our previous study [5]. Samples containing equivalent amounts of protein were loaded onto 10% to 15% acrylamide denaturing gels (sodium dodecyl sulfate polyacrylamide gel electrophoresis; SDS-PAGE). Membranes were probed overnight at 4°C with antibodies against Fas (sc-736), FasL (sc-6237), P2X_7_ (sc-25698), High mobility group box 1 (HMGB1, sc-26351), caspase-8 (sc-7890) (Santa Cruz Biotechnology Inc., Santa Cruz, CA, USA); CD3 (Abcam Cambridge, UK, Catalog no: ab5690); FADD (Epitomics, Inc., Burlingame, CA, USA, Catalog no: 2988–1); and β-actin (Sigma Chemical, St Louis, MO, USA, Catalog no:A2228). Immunoreactive proteins were visualized using an enhanced chemiluminescence detection system (Amersham Life Science, Buckinghamshire, UK).

### Fluoro-jade C staining

Fluoro-Jade C is a fluorescein derivative that has been shown to specifically stain degenerating neurons. In accordance with previous studies [33], free-floating sections were immersed in a 0.06% potassium permanganate solution for 10 min and then rinsed in distilled water for 1 min. The slides were then transferred to Fluoro-Jade C (Millipore) staining solution (0.001%) for 10 min. After staining, the sections were rinsed three times for 1 min each in distilled water.

### Immunohistochemical labeling, image acquisition, and analysis

Animals were anesthetized and transcardially perfusion-fixed with 4% paraformaldehyde in phosphate-buffered saline (PBS). Whole brains were immediately removed and postfixed overnight at 4°C in the same fixative. To identify distribution of microglial nodule in the brain, paraffin sections (4 μm) were stained with hematoxylineosin (H&E). For immunolabeling, coronal brain sections at the coordinates of anterior or posterior to bregma (35 μm in thickness) were prepared using a microslicer system (Vibratome, St Louis, MO, USA). Then, brain sections were incubated at room temperature with 0.01% Triton-X-100 in PBS for 30 min, followed by incubation in 3% bovine serum albumin (BSA) in PBS for 1 h. For immunolabeling, sections were incubated with antibodies to FasL (sc-6237) and P2X_7_ (sc-25698) (Santa Cruz Biotechnology Inc., Santa Cruz, CA, USA); GFAP (Sigma Chemical, St Louis, MO, USA, Catalog no: G3893); ED1 (MAB1435) and NeuN (MAB377) (Chemicon, Temecula, CA, USA); and Iba1 (Abcam Cambridge, UK, Catalog no: ab5076) overnight at 4°C, followed by exposure to fluorescent secondary antibodies according to a standard protocol from PerkinElmer Life Sciences, Inc. (Boston, MA, USA). Immunofluorescent images were taken with a confocal laser scanning microscope, and fluorescent density was analyzed using Image J software (NIH, Bethesda, MD, USA).

### Statistical analysis

The data are represented as the mean ± SEM. Statistical significance was determined using one-way analysis of variance (ANOVA) followed by Dunnett’s test for multigroup comparisons. A non-parametric Mann–Whitney U-test was used for analysis of the neurologic deficit data. *P* <0.05 indicated statistically significant differences.

### Results

#### Microsphere embolism-induced damage to microvessels caused a focal response in neighboring microglial cells

**Figure 1:**
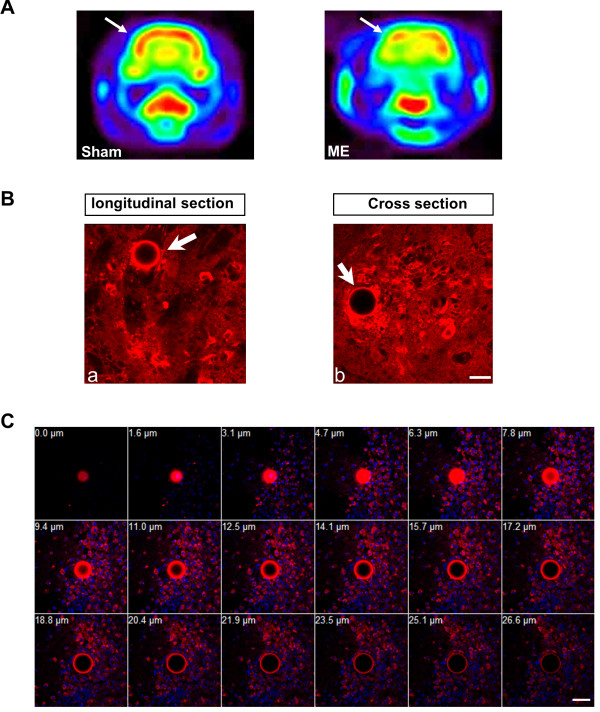
**Microsphere embolism-induced damage to microvessels caused a focal response in neighboring microglial cells.** (**A**) Changes in metabolic activity of glucose 168 h after microsphere embolism (ME) imaged on the microPET scanner. Images were taken from the same scan and reconstructed using the maximum a posteriori probability algorithm. Arrows indicates the ipsilateral hemisphere of rat brain. (**B**) Fluorescent staining with anti ED1 antibodies indicates predominant microglia activation beside the region blocked by microspheres (arrow). A number of activated microglia appeared to associate with a more distal region of the vessel as shown in the longitudinal section (a) and cross section (b). (**C**) Activated microglia were also found surrounding microsphere region (outlined in red), is shown in a series of XZ reconstructions. DAPI counterstaining indicates cell nuclei (blue). Scale bar = 50 μm. ME, microsphere embolism.

Microsphere embolism is a useful model to mimic the pathological process of human stroke or multi-infarct dementia; injected microspheres with appropriate size and number can specifically elicit microcapillary embolization rather than the larger artery injury [4-6]. Figure 1A shows that microsphere embolism induced decreasing of metabolic activity of glucose at 168 h after microspheres injection by using the microPET scanner. We also attempted to address the relationship between microsphere embolism and microglial activation in the ipsilateral hemisphere. After 168 h, we observed ED1-positive cells with ameboid morphology surrounding occluded microvessels in the rats with microsphere-induced embolism (Figure 1B and 1C).

### Temporal changes in FasL levels in rats following microsphere embolism

**Figure 2:**
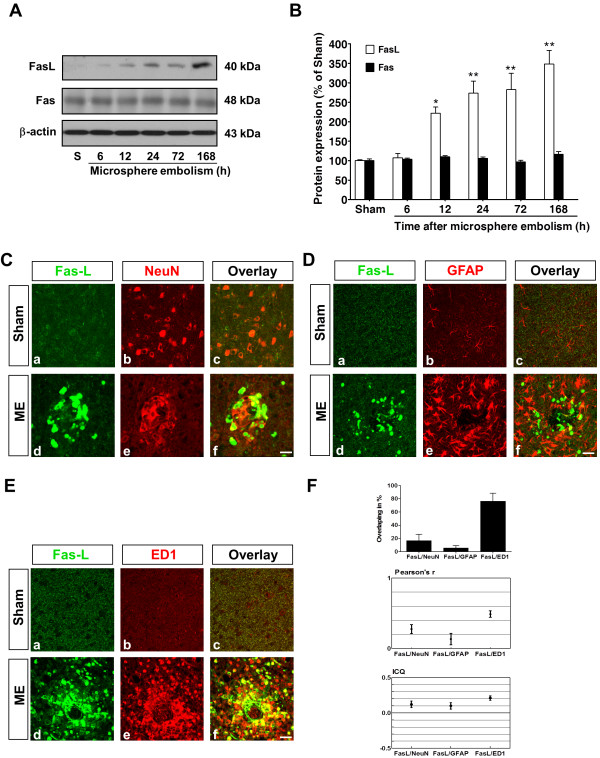
**Changes in FasL expression after microsphere embolism induction in the brain.** (**A**) Representative immunoblots showing Fas and FasL expression at the indicated times following microsphere embolism in rats. Immunoblotting with an anti-β-actin antibody showed equal total protein loading in each lane. S, sham. (**B**) Quantitative analysis of Fas and FasL levels was performed by densitometric analysis of the immunoblots. The data are expressed as percentages of values obtained from sham animals (mean ± SEM, * n * = 6 rats). * P  <0.05; **P <0.01 *vs.* sham. (**C**) Fluorescent staining for FasL (green) and NeuN (red) was performed in ipsilateral brain regions 168 h after embolic injury in rats. (**D**) FasL (green, a, d) and GFAP (red, b, e) double staining was performed 168 h after embolic injury in rats. (**E**) Immunohistochemical localization of FasL (green) and ED1 (red) was examined 168 h after ME ischemia. Scale bar = 40 μm. (**F**) Quantification of colocalized FasL-NeuN, FasL-GFAP, and FasL-ED1 fluorescent signals in rats following ME injury. The mean overlapping fraction, the mean Pearson’s r and the mean ICQ are presented for each condition (the mean ± SEM, *n* = 6 rats). ME, microsphere embolism.

FasL-Fas system is key initiators of the inflammatory response to injury [11]. To determine the roles of the Fas and FasL in microsphere-induced embolism, we initially used immunoblotting to investigate the temporal profile of FasL/Fas protein expression levels following microsphere embolism. A 40-kDa FasL immunoreactive protein band was detected by SDS-PAGE (Figure 2A). Representative blots are presented in Figure 2 and show a significant increase in FasL protein after 12 h in ME rats compared with sham rats (222.1 ± 15.6% *vs.* sham, *P* <0.05; Figure 2A and 2B). Further increases in FasL were evident after 168 h in the rats with microsphere embolism compared with the sham rats (348.1 ± 35.4% *vs.* sham, *P* <0.01). However, no significant differences in Fas expression were found (48 kDa) throughout the experiments (Figure 2A and 2B).

### FasL production is associated with microglial activation following microsphere embolism

To identify which cell types could potentially mediate the observed FasL signaling, we used double immunostaining for cell-specific markers to examine the cellular localization of FasL 168 h after the injection of microspheres. As shown in Figure 2C, the distribution patterns of FasL deposits and NeuN-positive staining were significantly different. In sham rats, the injection of 20% dextran did not induce an elevation in FasL expression (Figure 2C). A significant number of FasL-expressing cells, which were diffusely labeled with a round morphology, were found to be restricted to the occluding region 168 h after microsphere embolism (Figure 2C, d). In addition, astrocyte activation is known to be a significant component of postischemic pathophysiology [4]. A comparison of GFAP and FasL immunoreactivities demonstrated a near-complete dissociation of the staining patterns for these proteins in the ipsilateral hemisphere in ME rats (Figure 2D, d, e, f). Nearly all FasL-positive cells co-localized with ED1 (Figure 2E, f), whereas sham rats did not show significant positive staining for FasL or ED1 (Figure 2E, c).

The co-localization of fluorescent signals was analyzed using Image J software. NeuN and GFAP weakly co-localized with FasL-positive cells (FasL/NeuN, 16.4 ± 9.9%; FasL/GFAP, 5.5 ± 3.6%), whereas FasL strongly co-localized with ED1 (the mean overlapping fraction, 76.2 ± 12.3%; Figure 2F). Both Pearson’s r index and an intensity correlation coefficient (ICQ) analysis indicated similar results (Figure 2F). The present data indicate that robust FasL expression is predominantly associated with activated microglia after microsphere embolism in rats.

### Immunohistochemical localization of ED1- and Iba1-positive cells in the microembolic brain

**Figure 3:**
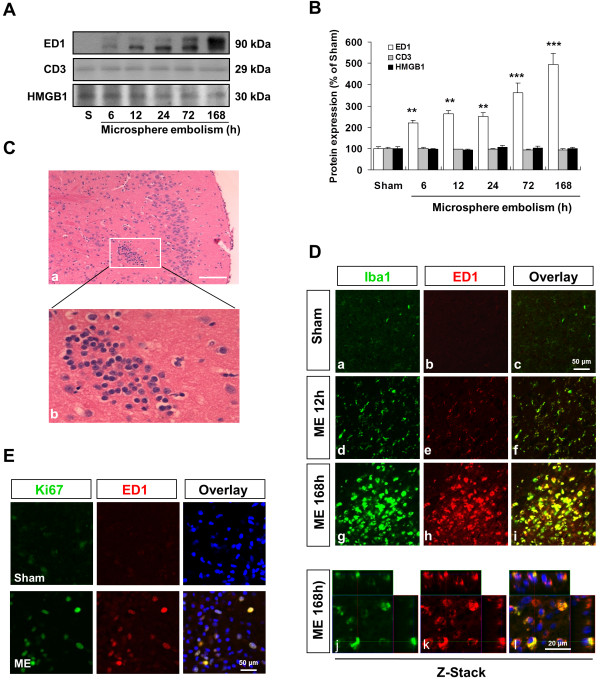
**Microsphere embolism induces microglia activation in brain of experimental animals.** (**A**) Temporal changes in ED1, HMGB1, and CD3 expression in ME rats. (**B**) Quantification of the normalized protein levels shown in (A). The data are expressed as percentages of values obtained from sham animals (mean ± SEM, *n* = 6 rats). **P <0.01; ***P <0.001 *vs.* sham. (**C**) H&E staining indicated that the presence of microglial nodules was noted ipsilateral to microsphere embolism. No infiltration of leukocytes, monocytes or macrophages was observed. Scale bar = 200 μm. (**D**) Immunohistochemical localization of ED1 (red)- and Iba1 (green)-positive cells in the microembolic brain. Iba1 labeling was used to identify the microglial population. Double-labeling studies revealed that Iba1 and ED1 staining were present in the same cell populations in areas of ME injury. Scale bar is 50 μm (a-i) and 20 μm (j, k, l). (**E**) Co-localization between the Ki-67 antigen and ED1-immunoreactive microglial cells in the ipsilateral region of brain. Scale bar = 50 μm. ME, microsphere embolism.

We further assessed longitudinal changes in ED1 expression after microsphere embolism. ED1 protein levels significantly increased in the brains of ischemic rats 6 h and 12 h (acute phase) after microsphere embolism compared with sham-operated rats. A secondary increase in ED1 was observed after 72 h, and the level remained elevated through 168 h (subacute phase) after microsphere embolism (Figure 3A, B). Consistent with previous reports, the early transient increase in ED1 might be associated with an adaptive immune response of microglial function to compensate for ischemic brain injury [34]. Indeed, we observed rapid activation of microglia in response to microembolic injury (apparent as early as 2 h after the event) that occurred with subsequent morphological changes similar to those observed in the late phase of microsphere embolism (data not shown). In the present study, the sources of lymphocytes and monocytes were examined using assays for CD3 and HMGB1, respectively. We did not find any significant differences in these markers after cerebral microemboli at any time point examined (Figure 3A, B). Concurrently, H&E staining indicated the presence of microglial nodules throughout the brain ipsilateral hemisphere to the microsphere embolism, whereas no infiltration of leukocytes, monocytes, or macrophages was observed (Figure 3C). Taken together, these data suggest that activated microglia might be the predominant source of FasL after microsphere embolism.

We also confirmed the specific activation of microglia during cerebral microemboli by immunostaining for Iba1, which is ubiquitously expressed in all microglial populations [35]. Double-labeling studies revealed that the number of Iba1/ED1-positive cells and the corresponding fluorescence intensity increased significantly in ME animals, including both ramified (12 h after microsphere embolism) (Figure 3D, d-f) and typical ameboid microglia (168 h after microsphere embolism (Figure 3D, g-i)). In addition, Iba1 and ED1 were present in the same cell populations in the ischemic region (Figure 3D, f and i). The observed irregularities in microglial cell shape support the possibility that the degree of morphological changes in Iba1/ED1-immunoreactive microglial cells is proportional to the duration of ischemic injury. The Ki-67 antigen is preferentially activated during all of the active phases of the cell cycle, but it is absent in resting cells. We found co-localization between the Ki-67 antigen and ED1-immunoreactive microglial cells in the ipsilateral brain region (Figure 3E).

### FasL elevation is associated with the migration of BV-2 microglia cells

**Figure 4:**
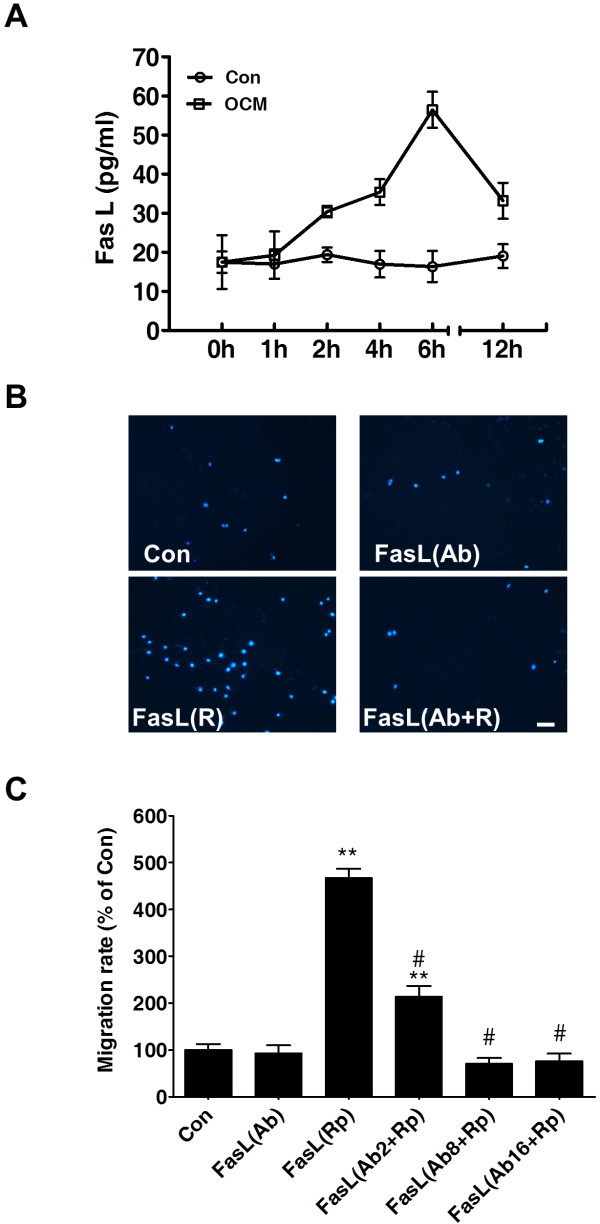
**FasL elevation is associated with the migration of microglia in BV-2 microglia cells.** (**A**) OCM treatment promotes FasL generation in BV-2 cells in a time-dependent manner. (**B**) Representative images of FasL-induced migration of microglia with or without the presence of FasL antibody. Scale bar = 100 μm. (**C**) Quantitative data of microglial migration following recombinant FasL treatment with or without FasL antibody pretreatment. **P <0.01 *vs.* Control; ^#^*P* <0.05 versus recombinant FasL treatment alone.

To investigate whether sustained FasL elevation is associated with the migratory capacity of microglia, we performed an *in vitro* transwell migration assay by using BV-2 cell line. In the present study, we found that FasL was rapidly elevated in response to OGD-treated cell-free conditioned medium (OCM) in the cultured BV-2 (Figure 4A) and peaked at 6 h after treatment (this peak was not observed in the control condition). Moreover, recombinant FasL (100 ng/mL) was added to microglial cultures, and the migration of cells were observed after a 24 h treatment with or without FasL antibody. The bar graph in Figure 4C summarizes the results from Figure 4B. These results demonstrate that recombinant FasL significantly promotes the migration of immortalized BV-2 microglia cells by 4.7-fold compared with the control cells (Figure 4A, B). Functionally, neutralizing FasL with blocking antibody was highly effective in inhibiting microglial mobility and chemotactic responses (FasL antibody 8 μg/mL, 70.69 ± 12.06% *vs.* Control; Figure 4C).

### P2X_7_ signal contributes to OGD-triggered immune responses by maintaining the overproduction of FasL in microglia cells

**Figure 5:**
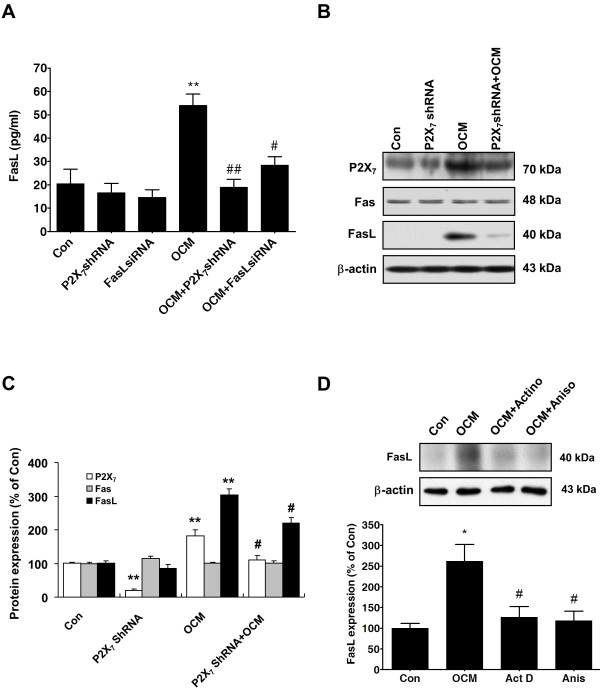
**P2X**_**7**_** signal contributes to the migration of microglia in BV-2 microglia cells.** (**A**) P2X_7_-specific shRNA transfection significantly reduced the increase in FasL generation induced by the OCM from OGD-stimulated cortical neurons. **P <0.01 *vs.* Control; ^#^*P* <0.05, ^##^*P* <0.01 *vs.* OCM treatment alone. (**B**) OCM-induced FasL protein expression was abolished by lentiviral infection of P2X_7_-shRNA in the BV-2 cell line. Quantified data are shown in (**C**). **P <0.01 *vs.* Control; ^#^*P* <0.05 *vs.* OCM treatment alone. (**D**) Representative immunoblots (upper) and quantitative analyses (lower) for FasL from BV-2 cultures following actinomycin D or anisomycin treatment. *P <0.05 *vs.* Control; ^#^*P* <0.05 *vs.* OCM treatment alone. OCM, OGD-treated cell-free conditioned medium.

In the brain parenchyma, injured cells release purines that initiate P2X_*7*_ purinergic signals, which act as early proinflammatory signals and lead to the production of cytokines and chemokines [36-38]. Therefore, we examined whether P2X_7_ expression could also regulate FasL production in cultured microglia, and we investigated the potential mechanisms associated with this process. Blockade of P2X_7_ signaling by P2X_*7*_-specific shRNA transfection significantly suppressed OCM-induced FasL production in the culture supernatants, whereas P2X_7_ shRNA alone did not affect FasL levels (Figure 5A), which indicated a causative role for P2X_7_ in initiating microglia-mediated FasL generation. Western blotting of BV-2 microglial cell lysates showed that microglia expressed FasL 6 h after the OCM treatment (Figure 5B, C). The suppression of P2X_7_ activation with P2X_7_ shRNA significantly prevented OCM-induced FasL expression but did not affect Fas expression (Figure 5B, C). These findings further support the idea that enhanced P2X_7_ expression may act as an important upstream regulator of FasL formation in OCM-treated microglial cells. These results highlight the ability of neuron-conditioned culture medium from OGD-stimulated cortical neurons to maintain a controlled inflammatory state through the activation of a P2X_7_-FasL signal that favors the microenvironment of activated microglia.

To ascertain whether OCM-induced increase in FasL protein required new mRNA and/or protein synthesis in microglial cells, we examined FasL levels in BV-2 cells treated with either the protein synthesis inhibitor anisomycin or the RNA polymerase inhibitor actinomycin D. We pretreated BV-2 cells with anisomycin or actinomycin D prior to treating them with OCM (Figure 5D). We found that the synthesis of FasL in microglia was sensitive to anisomycin treatment (Figure 5D). Concomitantly, actinomycin D pretreatment also decreased OCM-induced FasL elevation in BV-2 cells, indicating that OCM-induced FasL protein synthesis might dependent on new mRNA expression (Figure 5D). Thus, the present data suggest that OCM activates the local translation of FasL protein in a transcription-dependent manner. These results rule out the possibility that P2X_7_ knockdown might affect the general microglia protein translation machinery and suggest that P2X_7_ signaling is selectively involved in ischemia-induced microglial activation.

#### FasL is required for microglial migration

**Figure 6:**
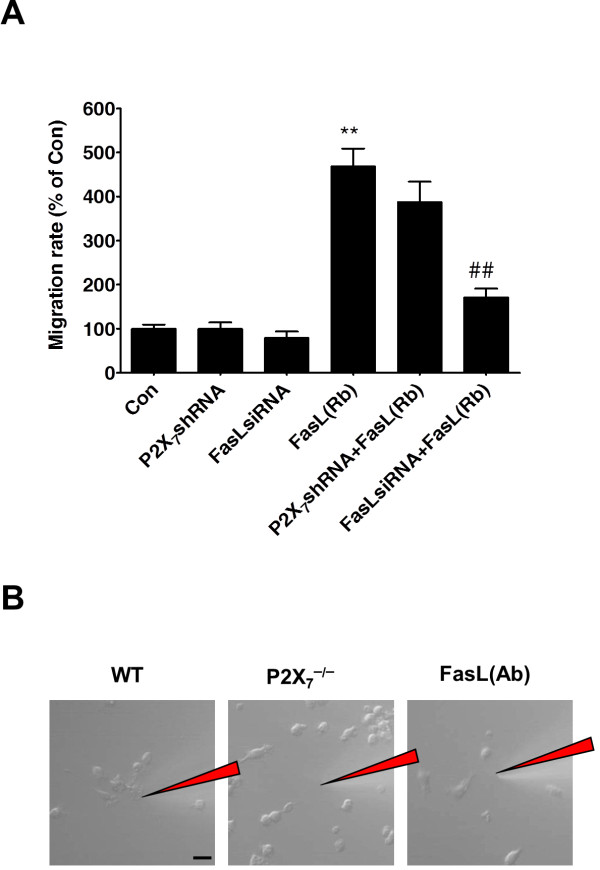
**FasL is required for microglial migration.** (**A**) A transwell assay was used to examine whether FasL silence or lentiviral infection of P2X_7_-shRNA could attenuate recombinant FasL-mediated microglial migration. **P <0.01 *vs.* Control; ^##^*P* <0.01 *vs.* recombinant FasL treatment alone. (**B**) FasL antibody neutralization or the loss of P2X_7_ receptors abrogates the response of microglia to ATP in primary microglial cell culture. Microglia from wild-type or P2X_7_^−/−^ mice visualized using time-lapse microscopy. Exuberant process extension toward a source point of ATPγS (1 mM from injection needle, red) was observed in the microglia of wild-type but not P2X_7_^−/−^ mice or preincubation with FasL antibody. Scale bar = 20 μm.

Moreover, a transwell assay was used to examine whether P2X_7_ plays a role in exogenous recombinant FasL-mediated microglial migration. We found that FasL siRNA transfection effectively reduced the number of BV-2 microglia that migrated toward the lower chamber relative to the control cells that only contained recombinant FasL (Figure 6A). However, P2X_7_ shRNA transfection did not significantly affect the exogenous recombinant FasL-mediated microglial migration (Figure 6A). These data further enforced the idea that P2X_7_ act as an upstream regulator of FasL production in activated microglial cells.

In light of the proposed roles for purinergic receptors in regulating microglial motility, we further investigated whether P2X_7_^−/−^ mice showed deficits in cellular responses to exogenous ATP. Upon application of Adenosine 5’-[γ-thio] triphosphate tetralithium salt (ATPγS, 1 mM), the vast majority of microglia from WT mice showed robust migration on time-lapse recordings (Figure 6B). In contrast, microglia cells from the P2X_7_^−/−^ mice did not show significant chemotactic response to ATP. Notably, preincubation with FasL antibody (8 μg/mL) significantly diminished the ATP-evoked chemotactic response of WT microglia *in vitro* (Figure 6B; see (Additional file 1: Movie 1, Additional file 2: Movie 2 and Additional file 3: Movie 3). Therefore, these results further suggest the critical functional roles for P2X_7_-FasL signaling in mediating microglial chemotactic inflammatory responses.

#### P2X_7_ is up-regulated in microglia after microsphere embolism

**Figure 7:**
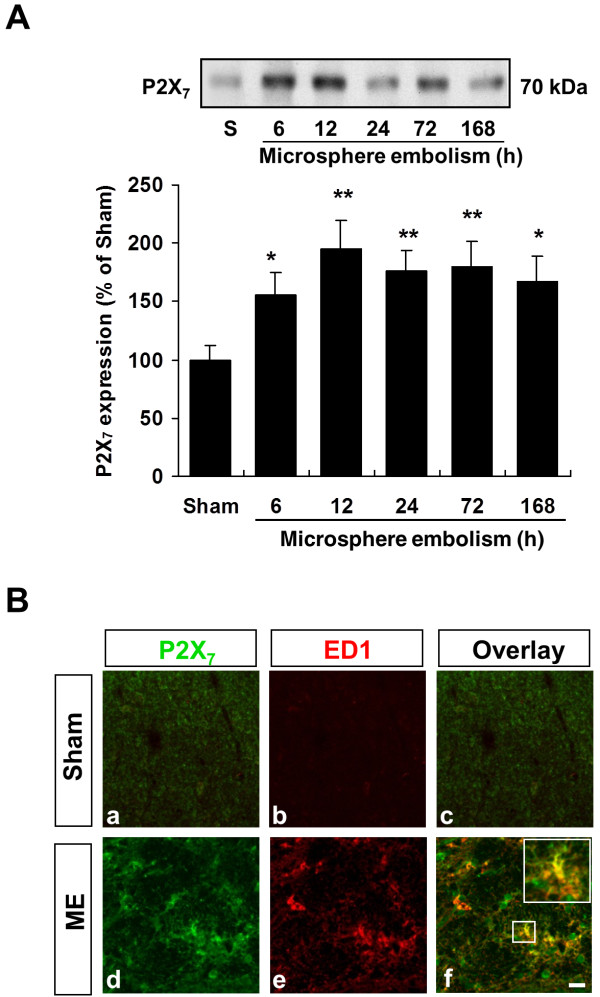
**P2X**_**7**_** participates in microsphere embolism-induced microglial activation.** (**A**) Temporal changes in P2X_7_ receptor expression levels after microsphere embolism induction in rat brain. The results are represented as the mean ± SEM, *n* = 6 rats. *P <0.05 and **P <0.01 *vs.* sham rats. (**B**) Immunohistochemical localization of P2X_7_- and ED1-positive cells in the microembolic brain. Scale bar = 20 μm. ME, microsphere embolism.

The experiments described above suggest that P2X_7_ is associated with the microglia-derived FasL overproduction. Therefore, we next investigated biochemical and morphological changes in P2X_7_ purinoceptors in cerebral microvascular occlusion models. Notably, we observed a dramatic and continuous increase in P2X_7_ immunoreactivity 6 h after microsphere embolism, which was consistent with the rapid activation of microglia (Figure 7A). Dual labeling of P2X_7_ and ED1 was used to ascertain whether the microsphere embolism-induced activation of microglia occurred in conjunction with P2X_7_ receptor-dependent signaling. Consistent with previous studies [39], we found that 168 h after microsphere embolism, the majority of P2X_7_ immunoreactivity localized to activated microglia (Figure 7B, d-f).

#### Microglia-induced neuronal injury in conjunction with P2X_7_ purinoceptor-FasL pathway activation

**Figure 8:**
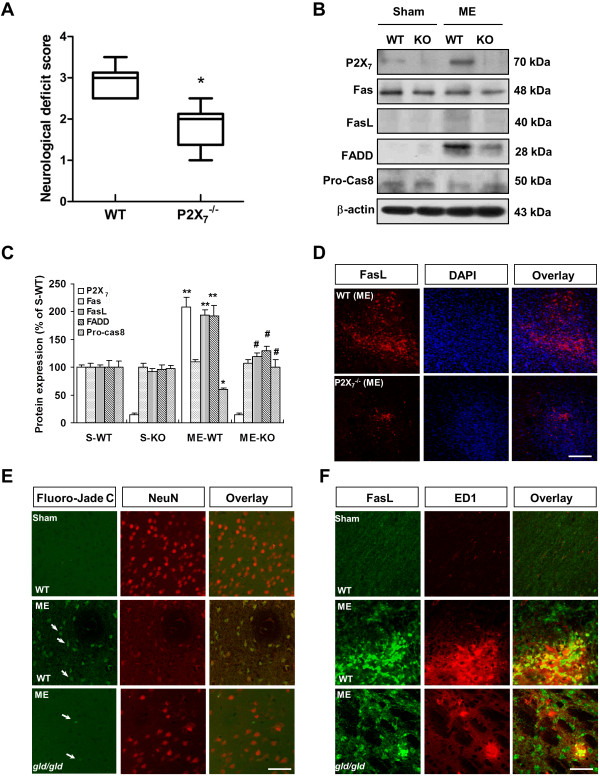
**Microglia-induced neuronal injury in conjunction with P2X**_**7**_**/FasL pathway activation.** (**A**) Neurological scores were determined 168 h after microsphere embolism in P2X_7_^−/−^ mice. The data are expressed as the mean ± SEM (*n* = 6 mice). *P <0.05 *vs.* WT mice. (**B**) Immunoblots demonstrating FasL/Fas cell death signaling measured 168 h after microsphere embolism in P2X_7_^−/−^ and WT mice. Quantified data are shown in (**C**). Data are expressed as means ± SEM (*n* = 6 mice). *P <0.05; **P <0.01 *vs.* Sham (WT); ^#^*P* <0.05 *vs.* ME (WT) group. S-WT, Sham (wild type). (**D**) Decreased FasL immunostaining was observed in P2X_7_^−/−^ mice 168 h after ME. DAPI counterstaining indicates cell nuclei (blue). Scale bar = 100 μm. (**E**) Fluoro-Jade C staining demonstrating the degree of neurodegeneration in WT and *gld/gld* mice 168 h after ME. Scale bar = 50 μm. ME, microsphere embolism. (**F**)**.** Immunohistochemical staining of FasL and ED1 in the ischemic brains of WT and *gld/gld* mice 168 h after ME. Scale bar = 50 μm.

The increase in P2X_7_ expression in microglia during microsphere embolism suggested a potential functional role for P2X_7_ in this process. To test this hypothesis, we subjected P2X_7_^−/−^ mice to 168 h of microsphere embolism. This time point was chosen because markers of cellular inflammation are robust in embolic injured brain at 168 h. Notably, neurological deficits were reduced in P2X_7_^−/−^ mice compared with strain-matched WT mice (Figure 8A). Consistent with these data, brains from the P2X_7_^−/−^ mice showed less induction of FasL, FADD, and caspase-8 after 168 h of microsphere embolism compared with WT controls (Figure 8B, C). In addition, less FasL-positive staining was observed in P2X_7_^−/−^ mice compared with WT mice (Figure 8D). The present study also implicated that elevated FasL levels are correlated with clinical severity of microsphere embolism.

To conclusively demonstrate the significance of FasL in ischemic injury *in vivo*, we also tested the neural sensitivity to cerebral microemboli insult in *gld/gld* mice. An increase in the number of Fluoro-Jade C-positive cells was observed in WT mice following microsphere embolism, whereas few Fluoro-Jade C-positive cells were present in FasL-deficient mice (Figure 8E). In addition, microsphere embolism significantly induced microglia activation and increased the number of FasL-positive cells in the brain ipsilateral hemisphere in WT mice, whereas both ED1- and FasL-positive staining were sparse in *gld/gld* mice 168 h after microsphere injection (Figure 8F).

#### ME-induced FasL activation and the apoptosis cascade are inhibited by minocycline treatment

**Figure 9:**
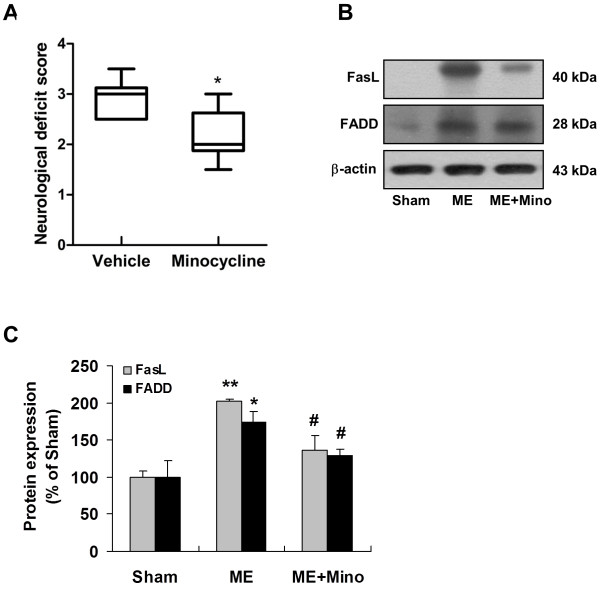
**Microsphere embolism-induced FasL activation and the apoptosis cascade were inhibited by minocycline treatment in ME rats.** (**A**) Neurological scores were determined 168 h after ME with or without minocycline treatment. Data are expressed as means ± SEM (*n* = 6 rats). *P <0.05 *vs.* vehicle-treated rats. (**B**) Immunoblots demonstrating FasL/Fas cell death signaling measured 168 h after ME with or without post-treatment with minocycline. Quantified data are shown in (**C**). Data are expressed as means ± SEM (*n* = 6 rats). *P <0.05; **P <0.01 *vs.* Sham; ^#^*P* <0.05 *vs.* ME group. ME, microsphere embolism.

Minocycline has emerged as a potent inhibitor of microglial activation, which is a member of the tetracycline antibiotic family [40,41]. It is interesting to consider whether peripheral administration of minocycline also affect microglia-derived increasing of FasL. As shown in Figure 9 (B, C), the protein level of FasL and FADD significantly increased 168 h after microsphere embolism, whereas prolonged minocycline treatment significantly inhibited elevation of FasL, which paralleled with the decreased FADD expression. These results further enforce that activation of microglia is linked with downstream FasL production in microglia. Concomitantly, we also found that prolonged minocycline treatment lead to improvement in neurological function (Figure 9A).

### Discussion

The present study addressed the pathophysiological relevance of cerebral microembolic injury-induced microglial activation, FasL intracellular signaling, and the neuronal death cascade. The primary findings of this study are three-fold. First, during the pathological process of cerebral microembolic injury, FasL signaling associated with ischemia-initiated microglial activation. As a key microenvironmental signal, overproduction of FasL further stimulated an extensive microglial response in an autocrine manner. Second, the effective inhibition of microglial activation was observed with P2X_7_ shRNA infection in cultured microglia or P2X_7_^−/−^ mice *in vivo*, and the inhibition of microglial activation was paralleled by inhibition of ischemia-induced FasL overgeneration. Third, permanent cerebral microemboli induced morphological and biochemical reactions in microglia that were consistent with neuronal apoptotic cascades. Based on the present findings, we hypothesize that modulation of microglial neurotoxicity may prove to be a potential therapeutic target in neurovascular diseases.

Several prior studies have revealed that microglia play a crucial role in mediating inflammatory responses following the initial insult [21,42-44]. Consistently, the present study demonstrates the rapid activation of microglia in response to microembolic injury, which is apparent as early as 2 h after the event. The robust FasL expression is predominantly associated with activated microglia in subacute phase of microsphere embolism. Several recent studies reported that purinergic receptors are involved in brain ischemia [39,45]. However, the relationship between microglial activation, FasL generation and neuronal death is not well understood and may involve multiple intracellular components [46-48]. In the present study, a rapid increase in P2X_7_ purinoceptor expression occurred in parallel with the early microglial activation in response to permanent microemboli injury, and P2X_7_ were predominantly expressed in ED1-expressing microglial cells. The P2X_7_ purinoceptor-mediated Ca^2+^ influx and activation of phospholipase C and the A2 cytoplasmic signaling pathways may be involved in proinflammatory cytokine release [36,49]. Here, the BV-2 cells with RNA interference and P2X_7_^−/−^ mice were used to further address the linkage between P2X_7_ and FasL overproduction. Our data revealed that FasL production was increased in the OCM-treated BV-2 cells, whereas P2X_7_ interference partially but significantly prevented this OCM-induced FasL overproduction. Similar results were also observed in P2X_7_^−/−^ mice with microsphere embolism *in vivo*. Monif *et al*. [50] reported that the overexpression of P2X_7_ purinoceptors was sufficient to drive microglial activation and morphological transformation. Therefore, we suggested that P2X_7_ purinoceptor may be an important upstream component of FasL generation, the activation of P2X_7_ in microglia contributes to a gradual accumulation of FasL along with the subsequent activation of downstream cascades.

The autocrine action of FasL-Fas system has been shown in vascular smooth muscle cells [14], lymphocytes [16], and leukemia K562 cells [51]. In the present study, the BV-2 cells and primary microglia from mice genetically deficient in P2X_7_ were used to further establish a linkage between microglia activation and FasL overproduction. Of particular relevance was an examination of microglial cultures with recombinant FasL treatment, which revealed that recombinant FasL significantly triggered microglia migration whereas was blocked by FasL antibody. In functional terms, we found that the levels of FasL generation agreed with the kinetics of migration of BV-2 cells. The microglial-mediated inflammatory autocrine responses involving cytokines are one important consideration in the pathology of neurovascular damage [52-54]. We suggested that the overproduction of FasL itself is sufficient functions as a key component of immunoreactive response loop by recruiting microglia to the lesion sites. Moreover, preincubation with FasL antibody significantly diminished the ATP-evoked chemotactic response of primary microglia *in vitro*. Therefore, we suggested that OGD-induced, microglia-derived FasL might be a key microenvironmental signal, which stimulated an extensive microglial response in ischemic context.

Tight regulation of microglial activation might be exploited to avoid unexpected and excessive cell death in the compromised brain [13]. We found that the deletion of P2X_7_ purinoceptors *in vivo* using P2X_7_^−/−^ mice significantly reduced the expression of FasL and concomitantly neuronal cell death in the embolic hemisphere of brain, which implicated that a constitutive elevation of FasL might act in concert with P2X_7_ to induce neurotoxicity. Mutations in Fas ligand are responsible for the single gene autoimmune *gld* phenotypes in mice [55]. Here, our data further indicate that *gld/gld* mice deficient in FasL were highly resistant to ischemia-induced neuronal damage. In addition, we found that minocycline treatment inhibited FasL signaling and subsequently reduced neuronal cell death in animals after microsphere embolism, which further suggested that microglia-derived FasL might contributes to a vicious cycle of neuronal cell death. Together, *in vivo* data also support the idea that P2X_7_ is involved in regulating the pathological immune response of microglia to microsphere-induced embolism via maintaining the activation of FasL.

The astrocyte-microglia collaboration forms a first-line innate immune mechanism in the parenchyma to detect mediators released from injured cells [56-58]. Whether FasL generation by activated microglia contributed to the activation of astrocytes in the present study, however, is a topic for further investigation. Additionally, when activated microglia change into a chronic profile following an injury, the cells either maintain their acute phenotype or divert into another activation state, may be chronically beneficial for neuronal survival and differentiation [10,59]. Therefore, to elucidate the role of P2X_7_/FasL signaling during chronic phase of ischemia should enable further progress to be made in the therapeutic translation of experimental observations to the bedside.

### Conclusion

**Figure 10:**
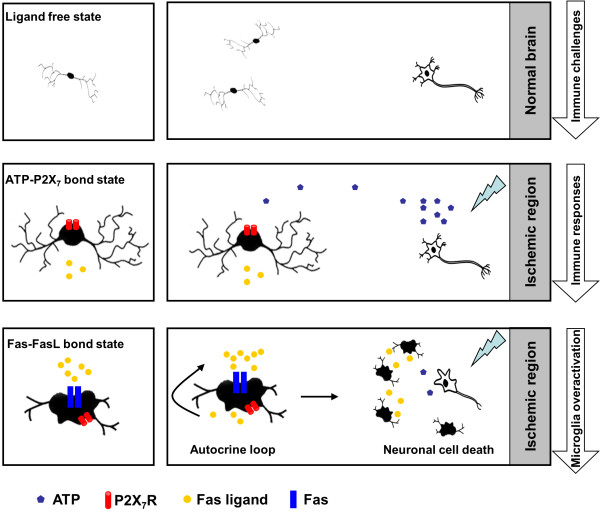
**Schematic illustration of the hypothesis of the FasL-mediated cell death by focal microglia activation via P2X**_**7**_**receptors during microsphere embolism.**

Based on our observations, the present study used the cerebral microemboli model and an *in vitro* culture system to provide further evidence of a role for microglial activation and P2X_7_/FasL-Fas signaling following ischemic insult. We demonstrate a new role for FasL in cerebral microvascular occlusion-mediated inflammatory responses and a previously unknown P2X_7_-based mechanism. In particular, these results also describe a mechanism that immunoreactive response loop of microglia-derived FasL further exaggerates P2X_7_-mediated microglial activation and triggers a vicious cycle of neuronal cell death in the ischemic context (Figure 10). The present results highlight the potential for a novel therapeutic neuroprotective approach using pharmacological blockade of sustained microglial activation and the death-inducing ligand in the acute and subacute phase of ischemia.

### Abbreviations

ATP, Adenosine 5-triphosphate; BSA, Bovine serum albumin; FADD, Fas-associated death domain; FasL, Fas ligand; GFAP, Glial fibrillary acidic protein; HMGB1, High mobility group box 1; OGD, Oxygen-glucose deprivation; SDS-PAGE, Sodium dodecyl sulfate polyacrylamide gel electrophoresis.

### Competing interests

The authors have declared that no competing interests exist.

### Authors’ contributions

FH, YML, and KF designed the research studies. YML, RRT, JYH, LTL, MHL, and ZHH performed the experiments. YML, XML, and FH drafted the manuscript with the assistance of the other authors. All authors have approved the manuscript.

## Supplementary Material

Additional File 1 **Movie 1 (For Figure 6B).** Migration of microglia from wild-type mice was recorded by time-lapse recording after exposure to a gradient of ATPγS (1 mM) for 30 min. Click here for file

Additional File 2 **Movie 2 (For Figure 6B).** Decreased migration of microglia from P2X7 knock out mice was recorded by time-lapse recording after exposure to a gradient of ATPγS (1 mM) for 30 min. Click here for file

Additional File 3 **Movie 3 (For Figure 6B).** Preincubation with FasL antibody (8 µg/mL) diminished the ATPγS (1 mM)-evoked migration of microglia from wild-type mice. Click here for file
